# Predictors of mortality among neonates in Lusaka, Zambia: a comparative analysis of machine learning and traditional survival analysis techniques

**DOI:** 10.3389/frai.2025.1606245

**Published:** 2025-11-11

**Authors:** Tshepiso Mokoena, Moses Mukosha, Moleen Zunza, Innocent Maposa

**Affiliations:** 1 Division of Epidemiology and Biostatistics, Faculty of Health Sciences, Stellenbosch University, Cape Town, South Africa; 2 Department of Pharmacy, University of Zambia, Lusaka, Zambia

**Keywords:** survival analysis, Weibull, machine learning, elastic net regression, neonatal mortality, predictive modeling

## Abstract

**Introduction:**

Neonatal mortality remains a critical global health issue, with 2.3 million deaths in 2022. Sub-Saharan Africa bears 57% of under five deaths despite only 30% of global births, with Zambia ranking fourth highest in terms of neonatal mortality among neighboring countries. While traditional survival analysis has identified neonatal mortality risk factors, machine learning-based prediction remains underexplored. This study aimed to identify factors associated with neonatal mortality and compare the predictive performance of traditional survival analysis and machine learning models among neonates in Lusaka, Zambia (January2018–September 2019).

**Methods:**

Demographic and clinical data from 1,018 neonates were analyzed using seven models: Weibull, Lasso, Ridge, Elastic Net (regularized Cox), Random Survival Forests, DeepSurv neural networks and Gradient Boosting Machines. Model performance was evaluated using nested cross-validation with five outer folds and three inner folds for hyperparameter tuning. Predictive accuracy was assessed using the concordance index, time dependent area under the curve at 7, 14, and 28 days, brier scores, and calibration plots. Kaplan–Meier plots illustrated survival probabilities over time.

**Results:**

Of the 1,018 neonates, 757 (74.3%) died. Hypoxic-ischemic encephalopathy (TR = 0.71, 95% CI: 0.63-0.81) was associated with reduced survival, while higher birthweight was protective (TR = 1.88, 95% CI: 1.60–2.20). Sepsis demonstrated a paradoxical association with longer survival (TR = 1.16, 95% CI: 1.04–1.30), which persisted in sensitivity analyses. Among predictive models, the Random Survival Forests achieved the highest discrimination (C-index = 0.731) and consistently low Brier scores, outperforming Weibull (C-index = 0.622) and penalized Cox models (≈ 0.620). Gradient Boosting Machines were most miscalibrated, and DeepSurv showed low discrimination (C-index = 0.553). Feature importance analysis from Random Survival Forest identified birth weight as the dominant predictor, followed by sex, sepsis, and necrotizing enterocolitis.

**Discussion:**

While traditional Weibull models remain valuable for interpretability, machine learning approaches provide enhanced predictive accuracy. Hybrid modeling strategies may improve early risk identification and inform neonatal care in resource-limited settings.

## Introduction

During the neonatal period, which encompasses the first 28 days of life, neonates are particularly vulnerable, facing the highest risk of mortality. In 2022, the global average rate of neonatal mortality stood at 17 deaths per 1,000 live births, marking a decrease from 18 deaths per 1,000 live births recorded in 2019 (UNICEF, 2021). The risk of mortality between the first month and the first year of life was estimated at 11 deaths per 1,000 live births, while the probability of mortality between ages 1 and 5 was estimated at nine deaths per 1,000 live births in 2022 (UNICEF, 2021). Globally, approximately 2.3 million infants lost their lives within the first month of birth in 2022. In the same year, sub-Saharan Africa had the world's highest neonatal mortality rate at 27 deaths per 1,000 live births and accounted for 57% of global under five deaths despite only 30% of global live births (UNICEF, 2021). Zambia has seen a notable improvement in its neonatal mortality rate, which has decreased to 24.1 deaths per 1,000 live births, from 25.4 in 2019 (Child Mortality and Causes of Death, 2024). Relative to surrounding countries such as, Zimbabwe (24.3), Namibia (18.7), Malawi (18.7), Mozambique (25.7), Angola (26.0), and Botswana (19.8), Zambia ranked 4th highest out of the six in 2022 (Child Mortality and Causes of Death, 2024). A range of policies, programs, and initiatives have contributed to Zambia's progress in reducing neonatal mortality. Key interventions include the Helping Baby Breathe campaign, Emergency Obstetric and Neonatal Care training, Saving Mothers Giving Life, and Safe Motherhood 360+ projects (Mukosha et al., 2021). These efforts are part of a broader strategy to achieve the Sustainable Development Goal (SDG) target of reducing neonatal mortality to 12 deaths per 1,000 live births by 2030 (Mukosha et al., 2021). Traditional statistical methods, particularly survival analysis have been a valuable tool in understanding neonatal mortality by analyzing time-to-event data, providing insights into the duration until an event, such as mortality, occurs. For instance, Sania et al. (2017)), utilized the Cox Proportional Hazards (CPH) model to examine the impact of birth weight and gestational age on neonatal mortality, revealing that lower birth weights and shorter gestational periods significantly increase the risk of mortality within the first 28 days. Similarly, Limaso et al. (2020)) utilized Kaplan–Meier survival curves and Cox regression to estimate the survival probabilities of neonates to emphasize the significance of early intervention and specialized care. In recent years, machine learning (ML) has emerged as a powerful tool for identifying patterns in large, complex datasets and predicting outcomes in various domains, including healthcare (Huang et al., 2023). However, its application in neonatal mortality remains limited, especially in low-resource settings like Zambia (Mukosha et al., 2021). A study by Cooper et al. (2018)) demonstrated the effectiveness of ensemble ML methods such as superlearning in predicting 30-day postoperative neonatal mortality, achieving excellent discrimination and outperforming individual regression models in predictive accuracy. This suggests that ML approaches could complement traditional survival analysis techniques in identifying neonates at highest risk of mortality. Neonatal mortality significantly impacts the quality of life for affected families and imposes a persistent burden on healthcare systems, leading to heavy economic and social costs. Accurate prediction of neonatal survival and understanding the factors driving these predictions are critical for targeted clinical interventions. Beyond the known risk factors for neonatal mortality, such as respiratory distress syndrome, neonatal sepsis, maternal health, prematurity, and congenital abnormalities as well as birth defects (Limaso et al., 2020; Gaiva et al., 2016; Chiabi et al., 2014; Zeinalzadeh et al., 2017), the study investigated additional factors such as birth weight and sex.

The study aimed to identify factors associated with neonatal mortality and to compare the predictive performance of traditional survival analysis with machine learning approaches, integrating both methods to enhance prediction among neonates in Lusaka, Zambia.

## Materials and methods

### Study design and population

This retrospective study used hospital records from 1st January 2018 to 30th September 2019, at the Women and Newborn Hospital in Lusaka, Zambia. The hospital is the largest referral hospital in Zambia for obstetric and gynecological care, receiving referrals from over 20 clinics and five first-level hospitals from surrounding areas of Lusaka and other parts of the country. Annually, the hospital manages approximately 28,800 pregnant women and records around 18,000 births, with over 4,000 neonatal admissions.

**Figure 1:**
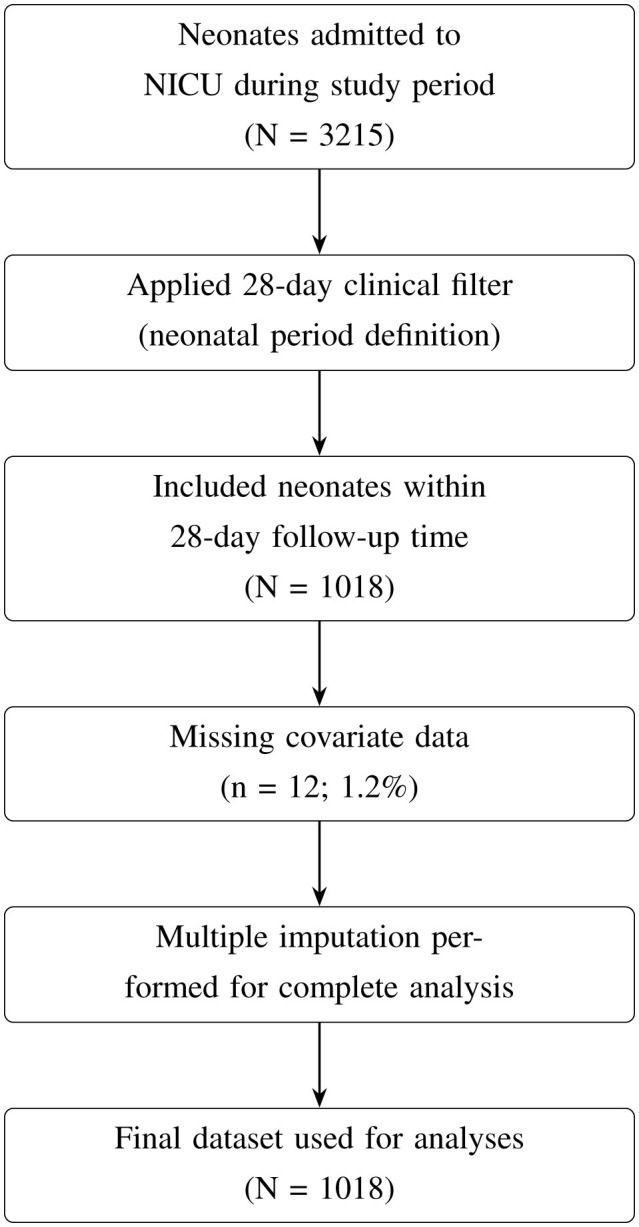
Flow diagram showing inclusion, exclusion, and imputation of study cohort.

This study focused on neonates admitted to the Neonatal Intensive Care Unit (NICU) or the Kangaroo Mother Care (KMC) ward. Data for this study were collected from Neonatal Case Records (NCR), which included demographic and clinical information on neonates and their mothers. Neonatal outcomes were followed from birth until discharge or death, with a complete 28-day follow-up period. The initial dataset included 3,215 neonates, which was filtered to include only those in the neonatal period, defined as the first 28 days of life (Figure 1). Neonates who survived beyond 28 days or were discharged before day 28 were right-censored, while those who died during hospitalization were classified as “died,” reflecting their survival status within the study window.

### Missing data handling

After applying the 28-day clinical filter, 1,018 neonates remained in the dataset. Of these, 12 neonates (1.2%) had missing covariate data, resulting in a final complete-case dataset of 1,006 neonates. Missing data were minimal across most variables (0.0%–0.3%), except for employment status (21.0%; Table 1).

**Table 1:** Missing data by variable.

**Variable**	**Missing *N***	**Total *N***	**Missing %**
Employment status	214	1,018	21.02
Hypoxic-ischemic encephalopathy	3	1,018	0.29
Necrotizing enterocolitis	2	1,018	0.20
Sepsis	2	1,018	0.20
Birth weight	2	1,018	0.20
Sex	1	1,018	0.10
Respiratory distress syndrome	0	1,018	0.00
Antenatal care visits	0	1,018	0.00
Mortality status	0	1,018	0.00
Time	0	1,018	0.00

Given the low levels of missingness, complete-case analysis was considered appropriate and unlikely to introduce meaningful bias. To further ensure robustness and preserve statistical power, multiple imputation using chained equations (MICE) was applied for variables with missing values in R using “mice” package. Binary and categorical variables were imputed using logistic regression, while continuous variables were imputed using predictive mean matching. Variables without missing data were included as predictors but were not imputed (Table 1).

A predictor matrix was specified to prevent imputation of the variables (time and status). Five imputed datasets were generated, and the first completed dataset was used for analyses. This approach ensured that the analysis leveraged all available data while accounting for minimal missingness.

**Justification for population selection:** the 68.3% reduction from the original dataset (3,215 → 1,018) is attributable to the 28-day clinical filter and reflects a deliberate focus on the neonatal period rather than data loss. This period captures the highest mortality risk and the most relevant clinical interventions for NICU patients.

In addition, only intra-hospital neonatal deaths were included. The dataset used in this study is not publicly available due to privacy concerns. Access to the dataset may be granted upon request to the data custodian.

The University of Zambia Biomedical Research Ethics Committee UNZABREC approved this study (ref: UNZA-221/2019). Additional permission was obtained from Women and Newborn Hospital management to extract data and conduct the study at the hospital. Data were de-identified to protect the participant's confidentiality. Furthermore, ethical approval was obtained requested from the research ethics committee at Stellenbosch University (Reference No: X24/06/016), with waiver of consent granted.

### Study variables

The outcome of this study was time to neonatal mortality, measured from birth to either mortality or censoring. Independent variables included human immunodeficiency virus (HIV) exposure, sex, sepsis, respiratory distress syndrome (RDS), necrotizing enterocolitis (NEC), hypoxic-ischemic encephalopathy (HIE), antenatal care (ANC) visits, and birth weight. These variables, along with their coding, are summarized in Table 2. Birth weight was categorized according to WHO standards of neonatal risk stratification as low (< 2.5 kg) or normal (≥2.5 kg) (WHO, 2022). For analysis, birth weight was included as a continuous variable in kilograms to retain full information and maximize statistical power. These variables were selected based on their clinical relevance and established associations with neonatal mortality, particularly in resource-limited neonatal settings.

**Table 2:** Description of variables included in the analysis.

**Variable**	**Coding**
Time	Time from birth to mortality or censoring (days)
Mortality status	Alive/died
HIV exposure	HIV-negative/positive
Sex	Male/female
Sepsis	Absent/present
Respiratory distress syndrome	Absent/present
Necrotizing enterocolitis	Absent/present
Hypoxic-ischemic encephalopathy	Absent/present
Antenatal care visits	Attended/no attendance
Birth weight	Continuous/categorical (low/normal)

### Statistical and machine learning considerations

The survival function, *S*(*t*), represents the probability of an event not occurring up to time *t*. Mathematically, it is expressed as:


$$\begin{array}{c} S\left(t\right)=P\left(T>t\right),\;0<t<\infty \end{array}$$

This function acts as a probability distribution over time, with *t* theoretically ranging from 0 to infinity, and *S*(*t*) values falling between 0 and 1 (Kleinbaum and Klein, 2012).

The survival function is typically visualized as a smooth, decreasing curve that starts at *S*(0) = 1 when *t* = 0, indicating that all subjects survive at the initial time (Kleinbaum and Klein, 2012).

An important characteristic of the survival function is that it is monotonically decreasing, meaning that:


$$\begin{array}{c} \left(S\left(t_{1}\right)>S\left(t_{2}\right)\right)\text{ when }\left(t_{1}<t_{2}\right) \end{array}$$

In practical terms, this indicates that the probability of surviving an event generally declines as time progresses (Kleinbaum and Klein, 2012).

Several models were developed to predict the survival outcomes based on the variables previously mentioned. The following models were implemented:

**Weibull accelerated failure time model:** the Weibull distribution is a commonly used parametric model in survival analysis as it generalizes the exponential model by allowing the hazard to vary over time. For survival time *t*>0, the hazard function, which represents the risk of event, is defined as (Kleinbaum and Klein, 2012):


$$\begin{array}{c} h\left(t\right)=\lambda pt^{p-1} \end{array}$$

From this definition, the survival function can be expressed as:


$$\begin{array}{c} S\left(t\right)=\exp\left(-\lambda t^{p}\right), \end{array}$$

where *t*>0, λ>0 is the scale parameter, and *p*>0 is the shape parameter (Kleinbaum and Klein, 2012).

Cases of the Weibull model include: when *p* = 1, the hazard is constant, which is equivalent to the exponential distribution; when *p*>1, the hazard increases over time; and when *p* < 1, the hazard decreases over time (Kleinbaum and Klein, 2012).

**Lasso, ridge, and elastic net regularized Cox models:** regularized Cox models include Lasso (α = 1), Ridge (α = 0), and Elastic Net (α = 0.5). The models perform variable selection and regularization by imposing an ℓ_1_, ℓ_2_ and ℓ_1_ and ℓ_2_ penalty on the coefficients respectively. This helps prevent overfitting and reduces model complexity by shrinking some coefficients to zero as well as reduce multicollinearity (Hastie et al., 2017).

The formula for regularized Cox regression introduces different penalties to the log-partial likelihood of the standard Cox model to perform variable selection or shrinkage. In the Lasso Cox model, a ℓ_1_ penalty term,


$$\begin{array}{c} \lambda \overset{p}{\underset{j=1}{\sum }}|\beta_{j}| \end{array}$$

is added to the objective function, encouraging sparsity by forcing some coefficients to zero, which effectively selects variables. In the Ridge Cox model, a ℓ_2_ penalty term,


$$\begin{array}{c} \lambda \overset{p}{\underset{j=1}{\sum }}\beta_{j}^{2}, \end{array}$$

is used instead, which shrinks coefficients toward zero but does not set any to exactly zero, making it useful for handling multicollinearity.

The Elastic Net Cox model combines both ℓ_1_ and ℓ_2_ penalties,


$$\begin{array}{c} \lambda \left(\alpha \overset{p}{\underset{j=1}{\sum }}|\beta_{j}|+\left(1-\alpha \right)\overset{p}{\underset{j=1}{\sum }}\beta_{j}^{2}\right) \end{array}$$

allowing for a balance between variable selection and shrinkage by tuning α (with α = 1 yielding the Lasso and α = 0 the Ridge). This flexibility makes Elastic Net advantageous in datasets with correlated predictors (Deng et al., 2023).

**Lasso Cox formula:**


$$\begin{array}{l} \hat{\beta }=\arg\underset{\beta }{\min}\{-\sum_{i=1}^{n}[\delta_{i}(\beta^{⊤}X_{i}\\ \;-\log(\sum_{j\in R(t_{i})}\exp(\beta^{⊤}X_{j})))]\\ \;+\lambda \sum_{j=1}^{p}|\beta_{j}|\} \end{array}$$

In this formula, $\hat{\beta }$ denotes the estimated coefficients that minimize the objective function. The first term represents the negative log partial likelihood from the Cox model, accounting for event indicators δ_*i*_ and the risk set *R*(*t*_*i*_). The second term, $\lambda \overset{p}{\underset{j=1}{\sum }}|\beta_{j}|$, is the Lasso penalty, where λ controls the strength of regularization and encourages sparsity in the coefficient estimates.

**Ridge Cox formula:**


$$\begin{array}{l} \hat{\beta }=\arg\underset{\beta }{\min}\{-\sum_{i=1}^{n}[\delta_{i}(\beta^{⊤}X_{i}\\ \;-\log(\sum_{j\in R(t_{i})}\exp(\beta^{⊤}X_{j})))]\\ \;+\lambda \sum_{j=1}^{p}\beta_{j}^{2}\} \end{array}$$

**Elastic Net Cox formula:**


$$\begin{array}{l} \hat{\beta }=\arg\underset{\beta }{\min}\{-\sum_{i=1}^{n}[\delta_{i}(\beta^{⊤}X_{i}\\ \;-\log(\sum_{j\in R(t_{i})}\exp(\beta^{⊤}X_{j})))]\\ \;+\lambda (\alpha \sum_{j=1}^{p}|\beta_{j}|\\ \;+\;(1-\alpha )\sum_{j=1}^{p}\beta_{j}^{2})\} \end{array}$$

**Random Survival Forests:** Random Survival Forests (RSFs) are an extension of the random forest method specifically designed for analyzing time-to-event data in survival analysis. As a tree-based ensemble model, RSFs construct multiple survival decision trees during training, capturing relationships between predictors and survival outcomes. Known for their robustness, random forests effectively handle high-dimensional data by averaging predictions from individual trees, which improves accuracy and reduces the risk of overfitting.

The RSFs regression:


$$\begin{array}{c} {\hat{f}}_{B}\left(x\right)=\frac{1}{B}\overset{B}{\underset{b=1}{\sum }}{\hat{f}}_{b}\left(x\right) \end{array}$$

In RSFs, base trees are typically grown to significant depths using bootstrapped samples, and the nodes are split by randomly selecting features. A key enhancement of RSFs is the incorporation of censoring information into the splitting criteria, enabling the model to create branches that reflect meaningful differences in survival outcomes. Various splitting criteria exist, such as conservation-of-events, log-rank score, and log-rank approximation; however, the log-rank splitting rule is the most widely used. This approach maximizes the log-rank test statistic, ensuring that the resulting splits reveal substantial disparities in survival between groups, thus enhancing the model's effectiveness in survival analysis (Hastie et al., 2017).

**Survival Neural Network:** Survival Neural Networks (SNNs) can feature one or more hidden layers connected to the preceding layer. Signals progress to the output layer, which is the final layer of units, producing the desired predictions (Hastie et al., 2017).

The SNN model we used in this study is based on DeepSurv, a type of Survival Neural Network specifically designed for survival analysis. DeepSurv is a neural network model that extends the CPH model by using a neural network to estimate the underlying hazard function, allowing for non-linear relationships between predictors and the hazard.

Key features of DeepSurv include its architecture, which consists of a feedforward neural network that takes covariates as inputs, passes them through one or more hidden layers, and outputs a single risk score analogous to the linear predictor in the Cox model. Its objective function minimizes the negative partial log-likelihood used in Cox models, thereby preserving the CPH framework (Wiegrebe et al., 2024).

DeepSurv can learn non-linear relationships, making it suitable for datasets with complex interactions among covariates. Additionally, DeepSurv offers flexibility in risk estimation through the inclusion of regularization techniques such as dropout and weight decay, which help to avoid overfitting; particularly beneficial for high-dimensional data.

SNN equations (Deng et al., 2023):

**Input layer:**


$$\begin{array}{c} x=\left[x_{1},x_{2},\ldots ,x_{n}\right] \end{array}$$

where *x* is the input vector containing features and *n* is the number of input features.

**Hidden layer:**


$$\begin{array}{c} z=\sigma \left(Wx+b\right) \end{array}$$

where *W* is the weight matrix, *b* is the bias vector, σ is the activation function and *z* is the output of the hidden layer.

**Output layer:**


$$\begin{array}{c} h\left(t;z\right)=\lambda \left(t\right)\cdot \exp\left(-\exp\left(z^{T}\beta \right)\cdot t\right) \end{array}$$

where *h*(*t*; *z*) is the hazard function at time *t*, λ(*t*) is the baseline hazard function, β is the parameter vector for the output layer.

**Loss function:** The loss function for training a survival neural network often involves the Cox partial likelihood:


$$\begin{array}{c} L=-\underset{i\in D}{\sum }\left(z_{i}^{T}\beta -\log\left(\underset{j\in R_{i}}{\sum }e^{z_{j}^{T}\beta }\right)\right) \end{array}$$

where *D* is the set of events, *R*_*i*_ is the risk set at time of event for individual *i*.

**Survival function:** The survival function can be derived from the hazard function:


$$\begin{array}{c} S\left(t\right)=\exp\left(-\int_{0}^{t}h\left(u;z\right)du\right) \end{array}$$

where *S*(*t*) is the survival function.

**Gradient Boosting Machines:** Gradient Boosting Machines (GBMs) extend naturally by minimizing a loss function adapted for censored data, most commonly the negative Cox partial log-likelihood. At each iteration *m*, a weak learner (typically a regression tree) is fit to the negative gradient of the loss, and the additive model is updated as:


$$\begin{array}{c} F_{m}\left(x\right)=F_{m-1}\left(x\right)+\nu \cdot h_{m}\left(x\right), \end{array}$$

where *F*_*m*_(*x*) is the cumulative risk score at iteration *m*, *h*_*m*_(*x*) is the base learner, and ν∈(0, 1] is the learning rate (shrinkage parameter). For the Cox model, the loss function is given by:


$$\begin{array}{c} L\left(\beta \right)=-\underset{i:\delta_{i}=1}{\sum }\left(\beta^{⊤}x_{i}-\log\underset{j\in R_{i}}{\sum }e^{\beta^{⊤}x_{j}}\right), \end{array}$$

where δ_*i*_ is the event indicator and *R*_*i*_ is the risk set at time *t*_*i*_. This framework allows GBMs to capture nonlinearities and interactions in survival data, but its sequential nature makes it prone to overfitting if the number of boosting iterations is too large, the learning rate too high, or trees too deep. Optimizing model performance requires careful tuning of shrinkage, sub-sampling, and cross-validation parameters (Bühlmann and Hothorn, 2007; Ridgeway, 2007).

### Statistical models

Descriptive statistics were used to summarize the baseline characteristics of the cohort using frequencies and percentages for all binary and categorical variables. Medians and inter-quartile ranges were reported for continuous variables. Kaplan–Meier overall survival curve was plotted to estimate survival probabilities against time.

#### Traditional survival methods

The CPH model was used as a baseline model in the study as a traditional survival analysis method. The Variance Inflation Factor (VIF) was used to assess multicollinearity. The VIF reflects how much the variance of the estimated coefficient for the variable is increased compared to what it would be if the predictor variables were uncorrelated. Values above 5 were noted as potential indicators of multicollinearity, suggesting the presence of redundant predictors. The proportional hazards assumption was formally tested using Schoenfeld residuals, examining both global and individual covariate tests. Variables violating the assumption were identified, and a stratified CPH model was fit by stratifying on the problematic covariates. However, the stratified model still violated the assumption, prompting the use of alternative parametric survival models. Two parametric models;Weibull and log-logistic accelerated failure time (AFT) models, were fit to account for the assumption violations. Model selection was guided by Akaike Information Criterion (AIC) and log-likelihood to assess model fit. Weibull model had a lower AIC value as well as higher log-likelihood values, and was therefore used as the final model. The Weibull model was assessed using a graphical check: Plot of log(–log(Survival)) vs. log(time); where a linear relationship indicates consistency with the Weibull assumption.

For both univariate and multivariate final models, coefficients were transformed into time ratios (TRs) for interpretability. Models were fit using the “survreg” function from the survival package in R(v4.4.0). For each predictor, we reported the estimated time ratio with corresponding 95% confidence intervals (CIs) and *p*-values. A TR > 1 indicates longer survival time, while a TR < 1 indicates shorter survival time (risk factor) (Hastie et al., 2017). A *p*-value of 0.05 was considered statistically significant.

### Machine learning models

A range of machine learning models was selected based on their use in survival analysis and ability to manage non-linear relationships, interactions, and censored data.

#### Model specifications and tuning

##### Regularized Cox models

The Lasso, Ridge and Elastic Net regularized Cox models were implemented via penalized maximum likelihood estimation using the “glmnet” package in R. This was to assess survival outcomes and perform variable selection. Nested cross-validation was used for hyperparameter tuning and model evaluation. Specifically, the dataset was split into *n* outer folds. Each outer fold served as a held-out test set, while the remaining folds were used for training. Within each outer training set, inner cross-validation was performed to select the optimal regularization parameter λ by minimizing the cross-validated partial likelihood. The Lasso model used an ℓ_1_ penalty (α = 1), the Ridge model used an ℓ_2_ penalty (α = 0), and the Elastic Net model used a combination (α = 0.5).

##### Random survival forests

RSFs were used to model survival outcomes using the “RandomForestSRC” package. For each outer fold of the nested cross-validation, the RSF model was trained on the outer training set, including all features and survival outcomes (time-to-event and censoring status). Each forest consisted of 100 trees to ensure stable estimates. Predictions for the held-out test set included the estimated mortality, which was used to calculate the C-index for survival discrimination. Short-term predictive performance was also evaluated with the area under the receiver operating characteristic curve (AUC) for 28-day mortality, computed only when a sufficient number of events was observed.

##### Survival neural networks

The DeepSurv architecture was implemented to model nonlinear relationships between features and survival outcomes. DeepSurv was fit using “survivalmodels” package. The network architecture included an input layer corresponding to the number of covariates, one or more hidden layers with rectified linear unit activation functions, and a linear output layer that produced risk scores proportional to the log-hazard function. To enhance generalizability and mitigate overfitting, several regularization techniques were applied. These included dropout regularization (with a dropout rate of 0.3), weight decay


$$\begin{array}{c} \lambda =10^{-4}, \end{array}$$

and early stopping with a patience of 50 epochs to monitor improvements in validation loss.

##### Gradient boosting machines

Nested cross-validation was used to optimize hyper-parameters and evaluate model performance. GBM was fit using “XGBoost” package. The dataset was split into *n* outer folds, with each fold serving as a held-out test set and the remaining folds used for training. Within each outer training set, inner cross-validation was employed to tune model-specific hyper-parameters. The number of boosting rounds was evaluated over candidate values (50, 100, 150), selecting the value that maximized the mean area under the AUC across inner folds.

The best-tuned model from each outer fold was trained on the full outer training set and used to generate predictions for the held-out test set, including linear predictors for survival discrimination (concordance index) and predicted probabilities for short-term classification performance for all models. To assess model stability and correct for overfitting, bootstrap resampling (*R* = 500) was performed. For each bootstrap sample, features were prepared as numeric matrices, models were refitted using the selected hyper-parameters, and the C-index was calculated. Ninety-five percent confidence intervals were derived from the percentile distribution of bootstrap C-indices, providing robust, optimism-corrected estimates of model discrimination and short-term predictive accuracy.

### Model evaluation metrics

After tuning and validation, the predictive performance of each model was assessed in terms of predictive survival times, discrimination, and calibration. Three key metrics were estimated;namely, the Concordance Index (C-index), Area Under the receiver operating characteristic (ROC) Curve and the Integrated Brier Score (IBS). In addition, calibration plots were presented.

#### Concordance index

By determining the percentage of correctly ordered patient pairs, the C-index assesses predictive accuracy of the predicted survival times and shows how closely projections match actual results. It is related to rank correlation techniques like Kendall's tau and may be used to a variety of outcomes, including continuous, binary, ordinal, and time-to-event (Park et al., 2021). A C-index of 0.5 in survival analysis indicates random predictions, but values near 1 signify perfect predictive power. This index aids in the efficient assessment of the model variables' predictive information (Park et al., 2021).

#### Time-dependent AUC

A common tool for assessing how well sensitivity and specificity are balanced in classification models is the receiver operating characteristic curve. As a classification rule's parameters are changed, it charts sensitivity against specificity. Stronger predictive performance is shown by greater AUC values, which quantitatively quantify the model's ability to discriminate between various outcomes, such as survival vs. event occurrence. The curve is expanded linearly to span [0, 100] to guarantee that it encompasses the whole range (Heagerty et al., 2000). For each time point, a binary outcome was created indicating whether an event occurred by that day. Time-dependent discrimination was assessed by comparing the linear predictor from the Cox model to this binary outcome using ROC analysis. To ensure reliable estimation, AUC was computed only when there were at least five events and at least five non-events at the respective time point. Bootstrap resampling with 500 iterations was applied to calculate 95% confidence intervals for the AUC values at relevant time points: 7, 14, and 28 days. These time points were selected to capture early, intermediate, and late outcomes, reflecting critical periods during which mortality events are most likely to occur and intervention decisions are often made.

#### Integrated brier score

Calibration of the models was assessed using the Brier score at fixed time points (7, 14, and 28 days), defined as the mean squared difference between observed event status and predicted probability of the event (Yang et al., 2022). Predicted probabilities at each time point were obtained from the Cox model using the baseline survival function and the individual's linear predictor:


$$\begin{array}{c} \hat{P}\left(T\leq t\right)=1-{Ŝ}_{0}{\left(t\right)}^{\exp\left(\text{lp}\right)} \end{array}$$

where Ŝ_0_(*t*) is the estimated baseline survival probability at time *t* and lp is the linear predictor. Predicted probabilities were truncated between 0 and 1 to avoid extreme values. To quantify uncertainty, bootstrap resampling was performed with 500 iterations. In each iteration, a bootstrap sample of the same size as the test dataset was drawn with replacement, and the Brier score was recalculated. The 95% confidence interval (CI) was then computed as the 2.5th and 97.5th percentiles of the resulting Brier score distribution.

#### Calibration analysis

Calibration of the predictive models was assessed at the same time points used for discrimination and Brier score evaluation (7, 14, and 28 days). Predicted risk scores were first normalized to a 0–1 scale then divided into deciles. Within each decile, the mean predicted probability and the observed event proportion were computed to summarize model calibration across low to high risk groups.

At each time point, a logistic regression of the observed binary event indicator on the predicted probabilities was performed to estimate the calibration slope and intercept. The calibration slope measures the agreement between predicted and observed risks, with a slope of 1 indicating perfect calibration (Austin et al., 2020). The intercept represents systematic over or underestimation of risk, with an ideal value of 0. Bootstrapped 95% confidence intervals of both slope and intercept were calculated to quantify uncertainty in calibration estimates.

Calibration plots were generated by plotting the mean predicted probabilities against the observed event rates across deciles. A 45-degree reference line representing perfect calibration was included, along with a smoothed loess curve. Facets were used to show calibration at each specified time point, allowing visual assessment of calibration over time.

## Results

### Baseline characteristics

Among the 1,018 neonates included in the analysis, 261 survived and 757 died. Maternal characteristics showed the median age of mothers whose neonates survived was 27 years (IQR: 22–33), compared to 26 years (IQR: 21–32) among those whose neonates died (Table 3). The slight difference may indicate that neonates born to younger mothers may have experienced higher mortality. The median birth weight among the cohort was 1.22 kg (IQR: 1.0–1.6 kg) among neonates who died, compared with 1.64 kg (IQR: 1.1–1.9 kg) among survivors (Table 3).

**Table 3:** Demographic characteristics by neonatal mortality.

**Variable**	**Overall (*N* = 1,018)**	**Died (*n* = 757)**	**Alive (*n* = 261)**	**Mortality rate (%)**
Birth weight (kg), median (IQR)	–	1.22 (1.0–1.6)	1.64 (1.1–1.9)	–
Age (years), median (IQR)	–	27 (22–33)	26 (21–32)	–
**Sex**				
Female	443	348	95	78.6
Male	575	409	166	71.0
**HIV exposure**				
No	822	622	200	75.7
Yes	196	135	61	68.9
**Necrotizing enterocolitis**				
No	962	712	250	74.0
Yes	56	45	11	80.4
**Sepsis**				
No	672	496	176	73.9
Yes	346	261	85	75.4
**Respiratory distress syndrome**				
No	554	398	156	71.8
Yes	464	359	105	77.4
**Hypoxic-ischemic encephalopathy**				
No	707	493	214	69.8
Yes	311	264	47	84.9
**Antenatal care visits**				
No	15	9	6	60.0
Yes	1003	748	255	74.6
**Employment status**				
No	936	596	153	63.7
Yes	82	44	11	53.7
**Birth weight category**				
Normal	361	216	145	59.8
Low	656	541	116	82.5

Although the number of deaths was higher among male neonates (*n* = 409) compared to female neonates (*n* = 348), the mortality rate was higher in females (78.6%) than in males (71.0%). This suggests that female neonates had a higher risk of mortality relative to the number of females in the study, indicating that female neonates in this cohort may have experienced worse survival outcomes compared to males.

Mortality was higher among HIV-negative neonates (*n* = 622) compared to HIV-positive neonates (*n* = 135). The mortality rate was higher in the negative group (75.7%) compared to the positive group (68.9%). This may indicate better clinical management of HIV-positive neonates. Neonates without necrotizing enterocolitis accounted for most deaths (*n* = 712), while neonates with the condition had fewer deaths (*n* = 45). However, the mortality rate was higher among neonates with necrotizing enterocolitis (80.4%) compared to those without (74.0%), suggesting a possible trend toward worse outcomes. Sepsis showed an unusual pattern, with similar mortality rates between affected neonates and those without sepsis, despite its clinical relevance as a common cause of neonatal mortality. A higher number of deaths was observed in neonates with sepsis (*n* = 260) than those without (*n* = 496). Mortality rates were comparable between the two groups, with 75.4% of mortality in neonates with sepsis and 73.9% in those without sepsis.

Mortality among neonates diagnosed with respiratory distress syndrome (*n* = 359) slightly exceeded those without the condition (*n* = 398). The mortality rate was higher in neonates with respiratory distress syndrome (77.4%) than those without (71.8%).

While the number of deaths was higher among neonates without hypoxic-ischemic encephalopathy (*n* = 493) compared to those affected (*n* = 264), the mortality rate was substantially higher in neonates with the condition (84.8%) compared to those without (69.8%). In addition, a higher number of deaths occurred in neonates whose mothers attended antenatal care (*n* = 748), compared to those whose mothers did not attend (*n* = 9). Neonates whose mothers did not attend antenatal care visits had a lower mortality rate (60.0%) than those whose mothers attended (74.6%). Lastly, neonates of unemployed mothers experienced more deaths (*n* = 596) than those of employed mothers (*n* = 44). Mortality rates were similar between the groups (63.7% vs. 53.7%, respectively).

### Bivariate analysis

Log-rank tests were conducted to compare survival distributions across several neonatal clinical and demographic variables (Table 4).

**Table 4:** Log-rank test results for survival by key variables.

**Variable**	**Chi-square**	***p*-value**
HIV exposure	0.1	0.7
Necrotizing enterocolitis	0.4	0.5
Sepsis	5.4	0.02
Respiratory distress syndrome	4.8	0.03
Hypoxic-ischemic encephalopathy	7.3	< 0.01
Sex	6	< 0.01
Birth weight	212	< 0.001
Antenatal care visits	0.9	0.3

There was no statistically significant difference in survival times between HIV-positive and negative neonates (χ^2^ = 0.1, *p* = 0.70), indicating that HIV exposure alone may not have a significant effect on neonatal survival in this cohort.

Survival differed significantly between neonates with and without sepsis (χ^2^ = 5.4, *p* = 0.02), indicating that the presence of sepsis is likely a determinant of mortality. Similarly, a significant association was observed between RDS and survival (χ^2^ = 4.8, *p* = 0.03), suggesting that RDS may negatively impact neonatal survival.

A strong association was found between hypoxic-ischemic encephalopathy and survival outcomes (χ^2^ = 7.3, *p* < 0.01), with neonates affected by HIE showing lower survival probabilities. Survival times differed significantly across birth weight categories (χ^2^ = 212, *p* < 0.001), confirming that low birth weight is a major determinant of neonatal mortality. A statistically significant difference in survival time was observed between male and female neonates (χ^2^ = 6.0, *p* = 0.01), suggesting that sex is associated with neonatal survival, with females experiencing lower survival probabilities.

Finally, no statistically significant difference in survival was observed based on antenatal clinic attendance (χ^2^ = 0.9, *p* = 0.30), suggesting that antenatal attendance alone may not significantly affect neonatal survival in this cohort.

### Univariate analysis

In the univariate analyses, several clinical factors were significantly associated with neonatal mortality risk (Table 5). Birth weight was a protective factor, with neonates of higher weight showing longer survival times (TR = 1.69, 95% CI: 1.47–1.95, *p* < 0.001). Being male was also significantly associated with 15% longer survival compared to females (TR = 1.15, 95% CI: 1.03–1.28, *p* = 0.01). In contrast, HIE was a significant predictor, associated with shorter survival (TR = 0.84, 95% CI: 0.75–0.94, *p* < 0.001).

**Table 5:** Univariate analysis.

**Predictor**	**TR**	**95% CI**	***p*-value**
HIV exposure	1.038	0.90–1.19	0.60
Sex	1.15	1.03–1.28	0.01
Antenatal care visits	0.95	0.58–1.55	0.83
Necrotizing enterocolitis	1.06	0.85–1.32	0.63
Sepsis	1.11	1.00–1.24	0.06
Respiratory distress syndrome	0.92	0.83–1.02	0.11
Hypoxic-ischemic encephalopathy	0.84	0.75–0.94	< 0.01
Birth weight	1.69	1.47–1.95	< 0.001

Among the other predictors, sepsis (TR = 1.11, 95% CI: 1.00–1.24, *p* = 0.06), HIV exposure status (TR = 1.04, 95% CI: 0.90–1.19, *p* = 0.60), ANC visits (TR = 0.95, 95% CI: 0.58–1.55, *p* = 0.83), NE (TR = 1.06, 95% CI: 0.85–1.32, *p* = 0.63), and RDS (TR = 0.92, 95% CI: 0.83–1.02, *p* = 0.11) were not significantly significant (Table 5).

### Multivariable analysis

After adjusting for all covariates, interestingly, sepsis was significantly associated with longer survival times (TR = 1.16, 95% CI: 1.04–1.30, *p* < 0.01), suggesting that neonates with sepsis survived approximately 16% longer compared to those without sepsis. Hypoxic-ischemic encephalopathy was associated with reduced survival, with affected neonates surviving 29% less time compared to those without HIE (TR = 0.71, 95% CI: 0.63–0.81, *p* < 0.001). Higher birth weight was a protective factor, associated with an 88% increase in survival time (TR = 1.88, 95% CI: 1.60–2.20, *p* < 0.001).

Other predictors, including HIV exposure (TR = 1.05, 95% CI: 0.91–1.20, *p* = 0.51), antenatal clinic visits (TR = 1.05, 95% CI: 0.65–1.71, *p* = 0.84), necrotizing enterocolitis (TR = 0.93, 95% CI: 0.74–1.16, *p* = 0.51), and respiratory distress syndrome (TR = 0.95, 95% CI: 0.84–1.07, *p* = 0.38), remained not significant after adjustment.

In contrast, sex (TR = 1.07, 95% CI: 0.96–1.19, *p* = 0.21) was no longer a significant predictor in the multivariate model, indicating that their initial univariate effect may be confounded by other clinical factors (Table 5).

### Graphical assessment of the Weibull assumption

**Figure 2:**
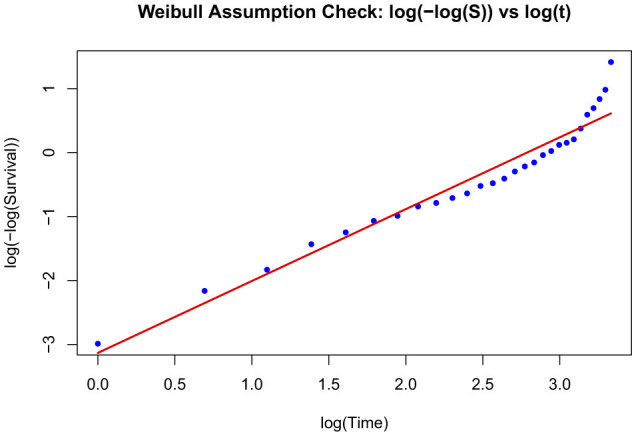
Plot of log(–log (survival)) vs. log(time).

The Weibull AFT model fit was assessed graphically using a log-minus-log transformation of the Kaplan–Meier survival estimates. The survival function *S*(*t*) was estimated for the entire cohort using the Kaplan–Meier method. To evaluate the appropriateness of the Weibull model, a plot of log(−log(*S*(*t*))) vs. log(*t*) was generated. A linear trend seen in this plot indicates that the Weibull distribution is a reasonable approximation for the survival times. A fitted regression line overlaid visually assesses linearity and confirm the suitability of the Weibull parametric model (Figure 2).

### Investigation of the sepsis paradox

To explore the apparent paradoxical association between sepsis and survival, in the multivariate model (TR = 1.16, 95% CI: 1.04–1.30, *p* < 0.01), a series of analyses was conducted. First, descriptive statistics were computed to summarize mortality rates and survival times by sepsis status. The distribution of mortality by sepsis status was examined. Table 6 shows that mortality was high in both groups: 496 of 672 (73.8%) neonates without sepsis died, compared to 261 of 346 (75.4%) with sepsis.

**Table 6:** Mortality by sepsis status.

**Sepsis**	**Survived**	**Died**
No sepsis	176	496
Sepsis	85	261

**Figure 3:**
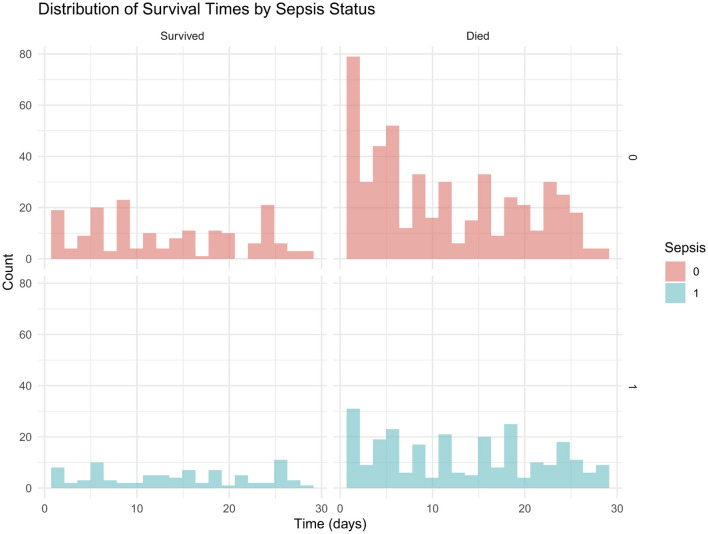
Sepsis status by survival status.

In addition, Figure 3 presents the distribution of survival times stratified by sepsis status, with histograms highlighting shorter survival among neonates with sepsis compared to those without.

Mortality rates and survival times by sepsis status are presented in Table 7. Mean survival times were similar between groups.

**Table 7:** Mortality rates and survival times by sepsis status.

**Sepsis**	***N***	**Deaths**	**Mortality rate**	**Mean (SD) survival time (days)**
0	672	496	0.738	5.2 (3.1)/4
1	346	261	0.754	5.0 (3.0)/4

#### Timing analysis: immortal time bias

To assess potential immortal time bias, early deaths (≤ 2 days) were compared between neonates with and without sepsis (Table 8). A lower number of early deaths occurred among those with sepsis (*n* = 180) compared to those without sepsis (*n* = 320).

**Table 8:** Early deaths (≤ 2 days) by sepsis status.

**Early death**	**No sepsis**	**Sepsis**
≤ 2 days	320	180
>2 days	176	81

This pattern suggests that some neonates classified as “non-sepsis” may have died before sepsis could be clinically diagnosed, potentially contributing to the paradoxical association of sepsis with longer survival observed in the multivariable analysis (Table 9).

**Table 9:** Multivariable analysis.

**Predictor**	**TR**	**95% CI**	***p*-value**
HIV exposure	1.05	0.91–1.20	0.51
Sex	1.07	0.96–1.19	0.21
Antenatal care visits	1.05	0.65–1.71	0.84
Necrotizing enterocolitis	0.93	0.74–1.16	0.51
Sepsis	1.16	1.04–1.297	< 0.01
Respiratory distress syndrome	0.95	0.84–1.07	0.38
Hypoxic-ischemic encephalopathy	0.71	0.63–0.81	< 0.001
Birth weight	1.88	1.60–2.20	< 0.001

#### Association with severity markers

Table 10 shows the association between neonatal sepsis and selected clinical severity markers. Sepsis was significantly associated with RDS, but not with HIE or NEC, indicating that neonates with sepsis were more likely to present with RDS, while the occurrence of HIE and NEC appeared independent of sepsis status.

**Table 10:** Association between sepsis and severity markers.

**Severity marker**	**Chi-square**	***p*-value**	**Cramér's *V***
Respiratory distress syndrome	16.2	< 0.001	0.13
Hypoxic-ischemic encephalopathy	3.6	0.06	0.06
Necrotizing enterocolitis	0.4	0.52	0.02

#### Stratified analysis by birth weight

Sepsis was stratified by birth weight (Table 11). Mortality remained high in all strata, but differences between sepsis and non-sepsis groups were reduced within strata. Among low birth weight neonates, mortality was comparable between those with sepsis (82%) and those without (80%). Similarly, in the normal birth weight group, mortality rates were nearly identical between sepsis (72%) and non-sepsis (71%) cases.

**Table 11:** Mortality by sepsis status, stratified by birth weight category.

**Birth weight category**	**Sepsis**	***N***	**Deaths**	**Mortality rate**
Low	0	200	160	0.80
Low	1	110	90	0.82
Normal	0	472	336	0.71
Normal	1	236	171	0.72

#### Weibull regression analyses

The unadjusted analysis suggested an association between sepsis and lower mortality (TR > 1), consistent with the observed sepsis paradox. Adjusting for birth weight and severity markers (RDS, HIE) revealed a more robust effect (TR = 1.16), indicating that the association in unadjusted models was partially due to confounding. Sensitivity analyses excluding early deaths (≤ 24 h and ≤ 48 h) produced similar TRs, suggesting that immortal time bias did not fully explain the paradox (Table 12).

**Table 12:** Time ratio estimates for sepsis from Weibull regression under different model specifications.

**Analysis**	**TR (sepsis)**	**95% CI**
Unadjusted	1.11	1.00–1.24
Adjusted (RDS, HIE, birth weight)	1.16	1.04–1.30
Excluding (≤ 24 h deaths)	1.09	0.99–1.21
Excluding (≤ 48 h deaths)	1.11	0.99–1.24

### Performance metrics

**Figure 4:**
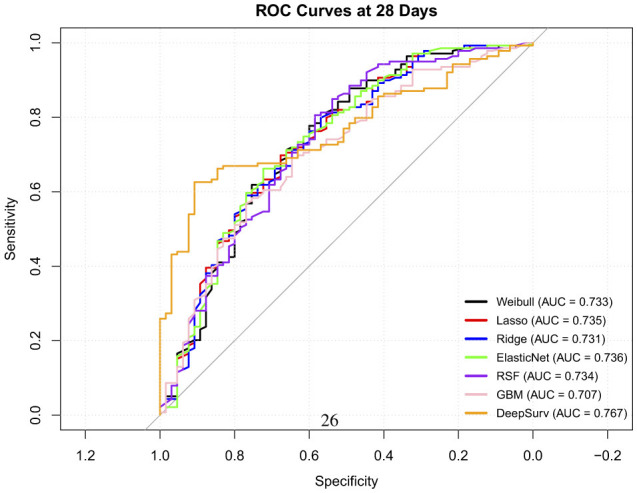
ROC curve at 28 days time point.

The time-dependent predictive performance of the seven models were compared (Table 13). The RSF model demonstrated the highest overall discriminative ability, with a C-index of 0.73 (95% CI: 0.72–0.748), outperforming all other models. Its time-dependent AUCs remained consistently strong across time points; 0.76 at 7 days, 0.66 at 14 days and 0.73 at 28 days (Figure 4), with low Brier scores (≤ 0.238), indicating good short and medium term calibration and reliable prediction accuracy. These findings highlight its ability to capture complex nonlinear relationships and interactions between predictors.

**Table 13:** Overall and time-dependent predictive performance metrics of the survival models.

**Model**	**C-index**	**95% CI**	**Time (days)**	**AUC**	**95% CI**	**Brier**	**95% CI**
Weibull	0.62	(0.60–0.65)	7	0.70	(0.62–0.77)	0.32	(0.29–0.35)
			14	0.65	(0.57–0.72)	0.33	(0.30–0.37)
			28	0.73	(0.66–0.81)	0.38	(0.35–0.41)
Lasso	0.62	(0.60–0.64)	7	0.68	(0.60–0.76)	0.27	(0.25–0.30)
			14	0.64	(0.56–0.72)	0.25	(0.22–0.28)
			28	0.73	(0.66–0.81)	0.20	(0.18–0.23)
Ridge	0.62	(0.60–0.64)	7	0.68	(0.59–0.76)	0.27	(0.25–0.30)
			14	0.64	(0.56–0.71)	0.26	(0.23–0.28)
			28	0.73	(0.65–0.80)	0.20	(0.18–0.23)
Elastic Net	0.62	(0.60–0.64)	7	0.68	(0.60–0.76)	0.26	(0.24–0.29)
			14	0.65	(0.57–0.73)	0.25	(0.22–0.28)
			28	0.74	(0.66–0.81)	0.21	(0.18–0.24)
RSF	0.73	(0.72–0.75)	7	0.76	(0.67–0.82)	0.20	(0.17–0.22)
			14	0.66	(0.59–0.74)	0.24	(0.22–0.26)
			28	0.73	(0.65–0.80)	0.21	(0.16–0.25)
GBM	0.60	(0.61–0.67)	7	0.68	(0.61–0.76)	0.47	(0.41–0.52)
			14	0.62	(0.54–0.70)	0.40	(0.34–0.45)
			28	0.71	(0.63–0.78)	0.22	(0.18–0.26)
DeepSurv	0.55	(0.40–0.58)	7	0.82	(0.76–0.88)	0.16	(0.14–0.19)
			14	0.60	(0.52–0.68)	0.26	(0.22–0.30)
			28	0.77	(0.70–0.83)	0.47	(0.43–0.51)

The Weibull model achieved moderate performance (C-index = 0.62; 95% CI: 0.60–0.65) with improving discrimination over time (AUC = 0.70 → 0.73) but relatively higher Brier scores, suggesting slightly poorer calibration. Similarly, the penalized Cox models (Lasso, Ridge, Elastic Net) showed comparable and stable performance across all time points (C-index = 0.620). Their AUCs improved from approximately 0.68 at 7 days to 0.73 at 28 days, accompanied by decreasing Brier scores approximately 0.27 to 0.20, indicating better long-term predictive accuracy but limited ability to model nonlinear relationships.

The GBM model achieved a modest C-index of 0.596 (95% CI: 0.61–0.67) and showed improvement in discrimination over time (AUC = 0.683 to 0.707), but it exhibited poor early calibration (7-day Brier = 0.47), suggesting over-fitting at shorter follow-up durations.

In contrast, DeepSurv yielded the lowest overall discrimination (C-index = 0.55; 95% CI: 0.40–0.58), with inconsistent time-dependent performance–excellent early discrimination (AUC = 0.82 at 7 days) but substantially reduced calibration at 28 days (Brier = 0.47). These fluctuations likely reflect model overfitting relative to the neural network's complexity.

Overall, the RSF model provided the best balance of discrimination and calibration across all time points, making it the most reliable for early risk stratification. Penalized Cox models offered interpretable and reasonably accurate alternatives, while the performance of GBMs and DeepSurv models may improve with larger datasets or hyperparameter tuning.

#### Calibration plots

**Figure 5:**
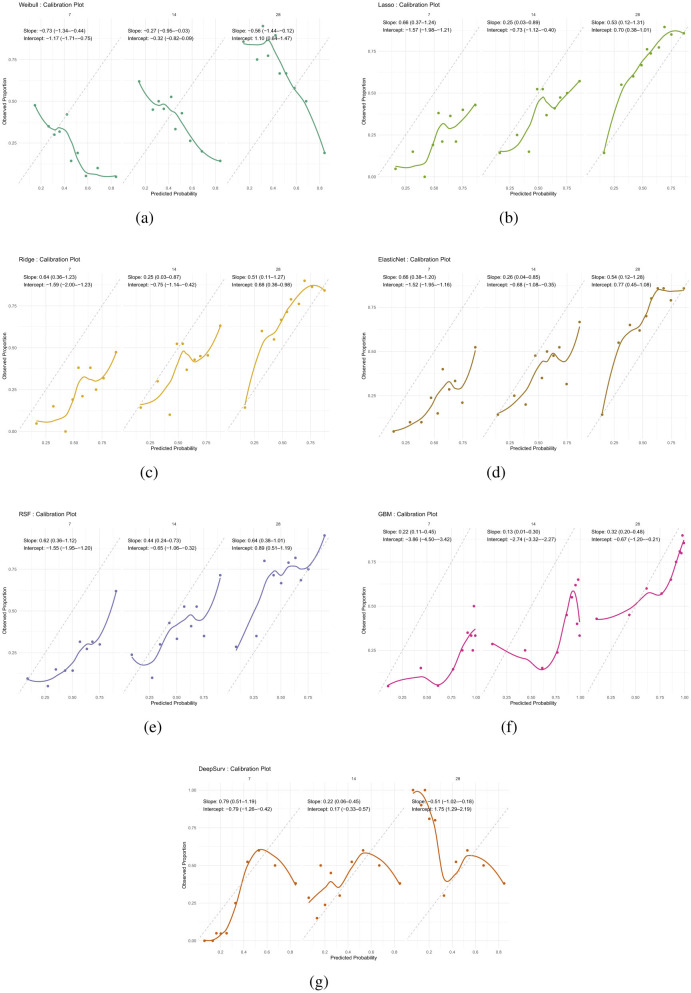
Calibration plots for all models at 28 days. Each plot compares predicted survival probabilities to observed outcomes. **(a)** Weibull. **(b)** Lasso. **(c)** Ridge. **(d)** Elastic Net. **(e)** RSF. **(f)** GBM. **(g)** DeepSurv.

Calibration analysis revealed distinct patterns of prediction bias across models and time points (Table 14). The Weibull model consistently overpredicted risk at early time points, with negative intercepts and slopes well below 1 (day 7: intercept –1.17, slope –0.73), indicating both systematic bias and overestimation. Although its intercept improved by day 28 (1.10), the slope remained suboptimal (–0.56), suggesting persistent miscalibration (Figure 5a).

**Table 14:** Calibration intercept and slope estimates with 95% confidence intervals across models and time points.

**Model**	**Time (days)**	**Intercept**	**95% CI**	**Slope**	**95% CI**
Weibull	7	–1.17	(–1.71 to –0.75)	–0.73	(–1.34 to –0.44)
	14	–0.32	(–0.82 to 0.09)	–0.27	(–0.95 to –0.03)
	28	1.10	(0.64 to 1.47)	–0.56	(–1.44 to –0.12)
Lasso	7	–1.57	(–1.98 to –1.21)	0.66	(0.37 to 1.24)
	14	–0.73	(–1.12 to –0.40)	0.25	(0.03 to 0.89)
	28	0.70	(0.38 to 1.01)	0.53	(0.12 to 1.31)
Ridge	7	–1.60	(–2.00 to –1.23)	0.64	(0.36 to 1.23)
	14	–0.75	(–1.14 to –0.42)	0.25	(0.03 to 0.87)
	28	0.69	(0.36 to 0.99)	0.51	(0.11 to 1.27)
Elastic Net	7	–1.52	(–1.95 to –1.16)	0.66	(0.38 to 1.20)
	14	–0.68	(–1.08 to –0.35)	0.26	(0.04 to 0.85)
	28	0.77	(0.45 to 1.08)	0.54	(0.12 to 1.28)
RSF	7	–1.55	(–1.95 to –1.20)	0.62	(0.36 to 1.12)
	14	–0.65	(–1.06 to –0.32)	0.44	(0.24 to 0.73)
	28	0.89	(0.51 to 1.19)	0.64	(0.38 to 1.01)
GBM	7	–3.86	(–4.50 to –3.42)	0.22	(0.11 to 0.45)
	14	–2.74	(–3.32 to –2.27)	0.13	(0.01 to 0.31)
	28	–0.68	(–1.20 to –0.22)	0.32	(0.20 to 0.48)
DeepSurv	7	–0.79	(–1.27 to –0.42)	0.79	(0.51 to 1.19)
	14	0.17	(–0.33 to 0.57)	0.22	(0.06 to 0.45)
	28	1.75	(1.29 to 2.19)	–0.51	(–1.02 to –0.18)

Regularized models (Lasso, Ridge, Elastic Net) showed similar behavior: intercepts changed from negative to positive over time, reflecting reduced bias, while slopes remained below 1, indicating overestimation. For example, Lasso's slope increased from 0.25 at day 14 to 0.53 at day 28, with intercepts shifting from –0.73 to 0.70. Ridge and Elastic Net followed comparable trajectories, with modest slope recovery and intercepts approaching zero by day 28 (Figures 5b–d).

RSF demonstrated progressive improvement, with intercepts moving from –1.55 to 0.89 and slopes from 0.62 to 0.64 across time points. While not perfectly calibrated, RSF showed the most consistent trend toward reliability (Figure 5e). In contrast, GBM exhibited miscalibration throughout: intercepts were negative (–3.86 at day 7), and slopes remained extremely low (0.13 at day 14), indicating overestimation and biased predictions (Figure 5f).

DeepSurv displayed the most favorable calibration at day 7, with intercept –0.79 and slope 0.79, closely approximating ideal values. However, its calibration deteriorated at later time points, with a reversed slope at day 28 (–0.51) and a high intercept (1.75), suggesting that its probability estimates were not consistent over time. Overall, while most models exhibited some degree of miscalibration, RSF and DeepSurv demonstrated comparatively better alignment between predicted and observed risks (Figure 5g).

#### Feature importance analysis

Since the RSF model was the overall best machine learning model, the importance of its features was interpreted (Table 15). The model demonstrated rapid convergence of error rates, stabilizing after approximately 300 trees, indicating adequate ensemble size for robust prediction. The analysis identified birth weight as the most important feature of neonatal survival, with a relative importance set to 1.00. This variable contributed substantially more to model performance than any other feature.

**Table 15:** Variable importance: random survival forest model.

**Variable**	**Importance**	**Relative importance**
Birth weight	0.15	1.00
Sex	0.05	0.33
Sepsis	0.04	0.29
Necrotizing enterocolitis	0.04	0.24
Hypoxic-ischemic encephalopathy	0.03	0.21
Respiratory distress syndrome	0.02	0.13
HIV exposure	0.02	0.12
Antenatal care visits	0.01	0.10

Neonatal sex, sepsis, and necrotizing enterocolitis emerged as the next most important predictors, each explaining roughly one-quarter to one-third of the predictive contribution of birth weight. Hypoxic-ischemic encephalopathy also demonstrated a meaningful effect, though of slightly lower magnitude. In contrast, respiratory distress, HIV exposure, and antenatal care visits were associated with lower importance values, indicating weaker contributions to overall model discrimination. These findings highlight the dominant role of birth weight in determining neonatal outcomes, while also highlighting the relevance of key clinical complications such as sepsis, necrotizing enterocolitis, and hypoxic-ischemic encephalopathy.

## Discussion

The high mortality rate (74.3%), reflects the vulnerability of this neonatal population and consistent with prior reports from low and middle income settings (Liu et al., 2016; Lawn et al., 2005).

Birth weight consistently remained as a strong predictor and confirmed the well established association between low birth weight and neonatal mortality (Lee et al., 2013). Neonates who died had a median birth weight of 1.22 kg compared to 1.64 kg among survivors. Although more male neonates died in numbers, the mortality rate was higher among females (78.6% vs. 71.0%), supporting prior reports of sex specific vulnerability (Kent et al., 2012), though the literature remains mixed (Mondal et al., 2014). The consistency of birth weight as a significant factor highlights its effect as a more robust marker of neonatal vulnerability. Conversely, a study in Botswana by Kitt et al. (2022)), found low birth weight to be protective factor. HIE was associated with reduced survival (TR = 0.71, *p* < 0.001), consistent with its known role in neonatal morbidity and mortality (Shankaran et al., 2005). RDS and NEC were associated with high mortality rates, though their effects were reduced in adjusted models, likely reflecting overlapping clinical pathways and limited sample size for NEC cases.

On the contrary, sepsis was associated with a reduced risk of mortality in adjusted models. This unusual finding challenges clinical assumptions and merits careful interpretation. A counterintuitive finding was that sepsis was associated with reduced mortality risk in multivariate models, with affected neonates surviving 16% longer than those without sepsis (TR = 1.16, 95% CI: 1.04–1.30). This “sepsis paradox” has been described in other critical care contexts (Seymour et al., 2016). Investigation showed that mortality rates were similar between septic and non septic neonates (75.4% vs. 73.8%), and Kaplan–Meier curves revealed overlapping survival distributions. Stratified analyses by birth weight reduced differences, suggesting confounding by growth restriction. Sepsis was also significantly associated with RDS, indicating clustering with other severity markers. Importantly, sensitivity analyses excluding early deaths (≤ 24 h and ≤ 48 h) yielded similar time ratios, suggesting that immortal time bias did not fully explain the paradox. Instead, the paradox may reflect that neonates who survived long enough to be diagnosed with sepsis also survived long enough to receive targeted treatment, therefore improving outcomes. This highlights the importance of considering timing of diagnosis and treatment effects in neonatal survival analyses (Fleischmann et al., 2021). In one large multi-center African analysis, a history of sepsis did not independently increase the risk of in-hospital neonatal death after adjusting for birth weight and prematurity (Ballot et al., 2010).

Factors such as the use of maternal antibiotics, negative repeat cultures within 72 h, and aggressive monitoring in neonatal care units may contribute to this observation. Singh's findings suggest that, in certain contexts, sepsis may signal a condition that has been rapidly identified and treated, leading to favorable outcomes (Singh et al., 2022). This aligns with observations by Batista et al. (2021)), emphasizing the positive impact of intensive neonatal care in enhancing outcomes. Though similar patterns were noted in Singh et al., the discussion in that context also lacked a critical synthesis.

Additionally, retrospective data often lack precise timing of diagnosis, illness severity, or treatment initiation, which limits accurate interpretation. Similar patterns have been observed in other retrospective cohorts (Singh et al., 2022), emphasizing the need for caution when interpreting unusual associations from observational data. Future research should incorporate time-varying variables as well as sepsis severity to better account for such biases in observational data.

Analysis demonstrated that the Random Survival Forest achieved the highest discriminative ability (C-index = 0.73), outperforming traditional Weibull AFT and penalized Cox models. This aligns with prior work showing that RSF effectively captures nonlinearities and interactions in survival data, often outperforming Cox-based approaches in biomedical applications (Ishwaran et al., 2008).

Recent neonatal survival studies also highlight the promise of machine learning. For example, Li et al. (2024)) demonstrated that Random Forest models improved prediction of survival in extremely premature neonates compared to traditional scoring systems. Similarly, external validation studies in high-mortality neonatal settings emphasize the importance of calibration alongside discrimination, showing that models often require updating to maintain accuracy across populations (Tuti et al., 2022).

Regularized regression models such as Elastic Net provided moderate discrimination but demonstrated stable calibration, consistent with their theoretical advantage in handling correlated predictors (Zou and Hastie, 2005). The DeepSurv model performed well initially but calibration and discrimination declined overtime, likely due to limited sample size, supporting prior findings that deep learning requires large-scale data to achieve stable generalization (Katzman et al., 2018). In addition, GBMs showed persistent miscalibration across all time points, reflecting over-prediction and poor probability scaling.

RSF feature analysis confirmed birth weight as the most influential feature. Neonatal sex, sepsis, and NEC followed. HIE also contributed meaningfully, while RDS, maternal HIV exposure, and antenatal care attendance were weaker predictors. Feature importance analyses in neonatal machine learning models have similarly highlighted birth weight, gestational age, and infection-related complications as key drivers of mortality risk (Li et al., 2024).

This study has several important limitations that must be acknowledged to contextualize the findings and interpret the results appropriately. First, because this was a retrospective study relying on routine clinical records, there's a possibility of bias due to data misclassification, missing variables that could not be measured, or simple inaccuracies. Notably, a large proportion of the original dataset, nearly 70% had to be excluded due to interest in specific variables, and ultimately based the analysis on complete cases only.

Secondly, the number of predictor variables used was limited to clinical variables and therefore had no access to time-updated clinical indicators such as vital signs or treatment changes.

Thirdly, the study was conducted at a single center, and it focused only on in-hospital outcomes. There was no follow-up on what happened to neonates after discharge, such as post-hospital mortality or readmission. This, combined with relatively short follow-up time, limits how broadly our findings can be applied to different settings or neonatal populations. In addition, although multiple imputation was performed, pooled estimates were not fully reported, and adherence to standardized reporting frameworks would strengthen reproducibility. The absence of time-specific data for sepsis also limited assessment of its temporal effects, which may explain the unexpected associations observed. Finally, the risk factors identified are rooted in the context of this particular neonatal group. It's entirely possible that these insights may not apply as well in other health systems or regions. Because of that, external validation in other hospitals, ideally across multiple centers and settings, will be essential. This kind of validation would allow assessment on whether findings in this study are robust, reproducible, and meaningful beyond this context. Despite these limitations, the results suggest potential integration points for ML in neonatal care. To bridge the gap between model development and clinical translation, future tools should prioritize both interpretability and predictive precision. Model transparency remains essential in sensitive clinical domains such as neonatal care, where decisions carry significant consequences.

## Conclusion

This study emphasizes the significant role of neonatal factors, particularly birth weight, sepsis and HIE in predicting mortality, with select socio-demographic factors such as sex also contributing to mortality risk. The comparison between Weibull and ML models highlights that while the Weibull model remains a reliable baseline model, RSF model demonstrated better predictive accuracy and feature selection flexibility. Ultimately, the choice of model should consider dataset size, variable model complexity, and clinical needs, as ML models, when used appropriately, can enhance predictive outcomes in neonatal health research. Future studies should explore hybrid approaches combining traditional and ML models to maximize both interpretability and predictive accuracy.

## Data Availability

The raw data supporting the conclusions of this article will be made available by the authors, without undue reservation.
